# Supplementary material for: Parasite defensive limb movements enhance acoustic signal attraction in male little torrent frogs
Source: eLife. 2022 May 6;11:e76083. doi: 10.7554/eLife.76083 (PMC9122496; doi:10.7554/eLife.76083)
Supplement: Supplementary file 1. — The data of total (spontaneous and parasite-induced) and parasite-induced displays in each limb movement for calling males, silent males, and males that have females nearby. [file elife-76083-supp1.docx]

**Table S1.** The data of total (spontaneous and parasite-induced) and parasite-induced displays in each limb movement for calling males, silent males and males that have females nearby.

| Number | Insect | Toe trembling | | Arm waving | | Wiping | | Limb shaking | | Leg stretching | | Foot flagging | | Hind foot lifing | | Noise (dB) |
| --- | --- | --- | --- | --- | --- | --- | --- | --- | --- | --- | --- | --- | --- | --- | --- | --- |
|  |  | total | insect | total | insect | total | insect | total | insect | total | insect | total | insect | total | insect |  |
| 1-Calling | 1 | 0 | 0 | 5 | 1 | 0 | 0 | 5 | 0 | 0 | 0 | 0 | 0 | 2 | 0 | 65.3 |
| 2-Calling | 8 | 0 | 0 | 8 | 6 | 1 | 1 | 4 | 1 | 0 | 0 | 0 | 0 | 2 | 0 | 72.3 |
| 3-Calling | 8 | 0 | 0 | 12 | 5 | 3 | 0 | 17 | 3 | 0 | 0 | 0 | 0 | 7 | 0 | 59 |
| 4-Calling | 11 | 0 | 0 | 19 | 9 | 1 | 0 | 17 | 2 | 1 | 0 | 0 | 0 | 7 | 0 | 57 |
| 5-Calling | 8 | 1 | 0 | 12 | 4 | 4 | 2 | 6 | 0 | 0 | 0 | 0 | 0 | 6 | 2 | 60.3 |
| 6-Calling | 25 | 0 | 0 | 2 | 1 | 2 | 2 | 40 | 18 | 3 | 0 | 0 | 0 | 8 | 4 | 58.5 |
| 7-Calling | 21 | 1 | 0 | 32 | 19 | 2 | 2 | 1 | 0 | 0 | 0 | 0 | 0 | 7 | 0 | 57 |
| 8-Calling | 5 | 0 | 0 | 5 | 3 | 0 | 0 | 1 | 1 | 2 | 0 | 4 | 0 | 7 | 1 | 70.3 |
| 9-Calling | 5 | 1 | 0 | 7 | 1 | 2 | 1 | 6 | 4 | 0 | 0 | 0 | 0 | 2 | 0 | 60 |
| 10-Calling | 34 | 0 | 0 | 36 | 30 | 2 | 1 | 6 | 3 | 0 | 0 | 0 | 0 | 8 | 1 | 57 |
| 11-Calling | 5 | 0 | 0 | 3 | 1 | 0 | 0 | 4 | 3 | 0 | 0 | 0 | 0 | 3 | 1 | 67.1 |
| 12-Calling | 9 | 0 | 0 | 4 | 2 | 5 | 1 | 12 | 6 | 0 | 0 | 0 | 0 | 1 | 0 | 53.4 |
| 13-Calling | 3 | 0 | 0 | 1 | 0 | 1 | 1 | 0 | 0 | 0 | 0 | 0 | 0 | 0 | 0 | 73.8 |
| 14-Calling | 0 | 0 | 0 | 0 | 0 | 0 | 0 | 6 | 0 | 0 | 0 | 0 | 0 | 5 | 0 | 75.8 |
| 15-Calling | 21 | 0 | 0 | 24 | 12 | 5 | 5 | 5 | 3 | 0 | 0 | 0 | 0 | 2 | 1 | 66.7 |
| 16-Calling | 9 | 0 | 0 | 7 | 3 | 3 | 0 | 18 | 5 | 0 | 0 | 0 | 0 | 4 | 1 | 69.8 |
| 17-Calling | 6 | 0 | 0 | 9 | 3 | 0 | 0 | 4 | 1 | 0 | 0 | 0 | 0 | 8 | 2 | 72 |
| 18-Calling | 4 | 0 | 0 | 2 | 1 | 2 | 2 | 1 | 0 | 0 | 0 | 0 | 0 | 1 | 1 | 70.5 |
| 19-Calling | 0 | 0 | 0 | 32 | 0 | 8 | 0 | 42 | 0 | 0 | 0 | 0 | 0 | 9 | 0 | 74.5 |
| 20-Calling | 1 | 0 | 0 | 26 | 0 | 5 | 1 | 9 | 0 | 0 | 0 | 0 | 0 | 10 | 0 | 69.7 |
| 21-Calling | 10 | 0 | 0 | 2 | 1 | 15 | 8 | 5 | 1 | 0 | 0 | 0 | 0 | 1 | 0 | 70 |
| 22-Calling | 4 | 0 | 0 | 7 | 0 | 1 | 1 | 5 | 1 | 0 | 0 | 0 | 0 | 3 | 2 | 66 |
| 23-Calling | 11 | 0 | 0 | 18 | 4 | 7 | 1 | 16 | 5 | 0 | 0 | 0 | 0 | 7 | 1 | 71.8 |
| 24-Calling | 33 | 0 | 0 | 52 | 20 | 10 | 7 | 15 | 3 | 0 | 0 | 0 | 0 | 13 | 3 | 58.5 |
| 25-Calling | 0 | 0 | 0 | 1 | 0 | 0 | 0 | 0 | 0 | 0 | 0 | 0 | 0 | 0 | 0 | 53.6 |
| 26-Calling | 48 | 0 | 0 | 20 | 17 | 8 | 6 | 43 | 24 | 0 | 0 | 0 | 0 | 5 | 1 | 74.1 |
| 27-Calling | 11 | 2 | 1 | 3 | 1 | 7 | 6 | 2 | 0 | 0 | 0 | 0 | 0 | 14 | 3 | 60.8 |
| 28-Calling | 0 | 0 | 0 | 1 | 0 | 1 | 0 | 9 | 0 | 0 | 0 | 2 | 0 | 3 | 0 | 76.9 |
| 29-Calling | 11 | 0 | 0 | 14 | 6 | 4 | 2 | 18 | 3 | 0 | 0 | 0 | 0 | 9 | 0 | 59 |
| 30-Calling | 5 | 0 | 0 | 3 | 2 | 2 | 1 | 5 | 1 | 0 | 0 | 0 | 0 | 1 | 0 | 67 |
| 31-Calling | 4 | 0 | 0 | 16 | 3 | 3 | 0 | 28 | 1 | 0 | 0 | 0 | 0 | 0 | 0 | 72.7 |
| 32-Calling | 0 | 0 | 0 | 1 | 0 | 1 | 0 | 2 | 0 | 0 | 0 | 0 | 0 | 0 | 0 | 59.5 |
| 33-Calling | 3 | 0 | 0 | 2 | 1 | 0 | 0 | 7 | 2 | 0 | 0 | 0 | 0 | 2 | 0 | 67.5 |
| 34-Calling | 4 | 0 | 0 | 3 | 0 | 2 | 0 | 18 | 3 | 0 | 0 | 4 | 0 | 0 | 0 | 68.3 |
| 35-Calling | 12 | 0 | 0 | 15 | 2 | 5 | 3 | 30 | 5 | 0 | 0 | 0 | 0 | 11 | 2 | 67.8 |
| 36-Calling | 30 | 7 | 0 | 22 | 6 | 8 | 3 | 58 | 20 | 0 | 0 | 0 | 0 | 2 | 1 | 58.3 |
| 37-Calling | 25 | 0 | 0 | 18 | 10 | 3 | 3 | 20 | 11 | 0 | 0 | 0 | 0 | 1 | 1 | 68.1 |
| 38-Calling | 5 | 0 | 0 | 1 | 0 | 3 | 2 | 14 | 3 | 0 | 0 | 0 | 0 | 1 | 0 | 57.3 |
| 39-Calling | 7 | 0 | 0 | 4 | 0 | 1 | 0 | 13 | 7 | 0 | 0 | 0 | 0 | 6 | 0 | 64.5 |
| 40-silence | 0 | 0 | 0 | 0 | 0 | 0 | 0 | 0 | 0 | 0 | 0 | 0 | 0 | 0 | 0 | 72.5 |
| 41-silence | 0 | 0 | 0 | 0 | 0 | 0 | 0 | 0 | 0 | 0 | 0 | 0 | 0 | 0 | 0 | 72.5 |
| 42-silence | 0 | 0 | 0 | 0 | 0 | 0 | 0 | 0 | 0 | 0 | 0 | 0 | 0 | 0 | 0 | 68.2 |
| 43-silence | 0 | 0 | 0 | 0 | 0 | 0 | 0 | 0 | 0 | 0 | 0 | 0 | 0 | 0 | 0 | 60.8 |
| 44-silence | 4 | 0 | 0 | 1 | 1 | 2 | 2 | 1 | 1 | 0 | 0 | 0 | 0 | 0 | 0 | 57 |
| 45-silence | 2 | 0 | 0 | 0 | 0 | 0 | 0 | 1 | 1 | 1 | 0 | 1 | 0 | 1 | 1 | 56.3 |
| 46-silence | 0 | 0 | 0 | 0 | 0 | 0 | 0 | 0 | 0 | 0 | 0 | 0 | 0 | 0 | 0 | 59.8 |
| 47-silence | 5 | 0 | 0 | 0 | 0 | 0 | 0 | 5 | 5 | 0 | 0 | 0 | 0 | 0 | 0 | 57.2 |
| 48-silence | 0 | 0 | 0 | 0 | 0 | 0 | 0 | 4 | 0 | 0 | 0 | 0 | 0 | 0 | 0 | 60.2 |
| 49-silence | 3 | 1 | 1 | 0 | 0 | 0 | 0 | 5 | 3 | 0 | 0 | 0 | 0 | 0 | 0 | 68.2 |
| 50-silence | 0 | 0 | 0 | 0 | 0 | 0 | 0 | 0 | 0 | 1 | 0 | 0 | 0 | 0 | 0 | 62 |
| 51-silence | 16 | 0 | 0 | 2 | 1 | 1 | 1 | 24 | 11 | 0 | 0 | 0 | 0 | 1 | 1 | 72 |
| 52-silence | 2 | 0 | 0 | 2 | 0 | 0 | 0 | 5 | 2 | 0 | 0 | 0 | 0 | 0 | 0 | 67.4 |
| 53-silence | 0 | 0 | 0 | 1 | 0 | 0 | 0 | 0 | 0 | 0 | 0 | 0 | 0 | 0 | 0 | 62.1 |
| 54-silence | 0 | 0 | 0 | 1 | 0 | 0 | 0 | 1 | 0 | 0 | 0 | 0 | 0 | 0 | 0 | 64.1 |
| 55-silence | 0 | 0 | 0 | 0 | 0 | 0 | 0 | 0 | 0 | 0 | 0 | 0 | 0 | 0 | 0 | 63.6 |
| 56-silence | 0 | 2 | 0 | 0 | 0 | 0 | 0 | 0 | 0 | 0 | 0 | 0 | 0 | 0 | 0 | 60.4 |
| 57-silence | 0 | 0 | 0 | 0 | 0 | 0 | 0 | 0 | 0 | 0 | 0 | 0 | 0 | 0 | 0 | 76.4 |
| 58-silence | 0 | 0 | 0 | 0 | 0 | 0 | 0 | 0 | 0 | 0 | 0 | 0 | 0 | 0 | 0 | 61.8 |
| 59-silence | 13 | 0 | 0 | 14 | 6 | 3 | 1 | 6 | 1 | 0 | 0 | 0 | 0 | 8 | 5 | 61.4 |
| 60-silence | 1 | 0 | 0 | 3 | 1 | 0 | 0 | 0 | 0 | 0 | 0 | 0 | 0 | 1 | 0 | 63.5 |
| 61-silence | 0 | 1 | 0 | 2 | 0 | 0 | 0 | 0 | 0 | 0 | 0 | 0 | 0 | 0 | 0 | 74 |
| 62-silence | 31 | 0 | 0 | 2 | 1 | 0 | 0 | 26 | 26 | 0 | 0 | 0 | 0 | 4 | 4 | 73.5 |
| 63-silence | 7 | 3 | 1 | 5 | 1 | 1 | 0 | 4 | 4 | 0 | 0 | 3 | 0 | 0 | 0 | 63.8 |
| 64-silence | 0 | 0 | 0 | 1 | 0 | 0 | 0 | 0 | 0 | 0 | 0 | 0 | 0 | 0 | 0 | 65 |
| 65-silence | 0 | 3 | 0 | 0 | 0 | 0 | 0 | 6 | 0 | 0 | 0 | 0 | 0 | 0 | 0 | 61 |
| 66-silence | 2 | 1 | 0 | 1 | 1 | 0 | 0 | 8 | 1 | 0 | 0 | 0 | 0 | 1 | 0 | 74 |
| 67-silence | 6 | 1 | 0 | 5 | 3 | 1 | 0 | 4 | 2 | 0 | 0 | 0 | 0 | 2 | 1 | 78 |
| 68-silence | 9 | 0 | 0 | 0 | 0 | 0 | 0 | 17 | 9 | 0 | 0 | 0 | 0 | 0 | 0 | 69.4 |
| 69-silence | 3 | 0 | 0 | 1 | 1 | 1 | 1 | 1 | 1 | 0 | 0 | 0 | 0 | 0 | 0 | 61 |
| 70-♀♂ | 0 | 0 | 0 | 0 | 0 | 0 | 0 | 0 | 0 | 1 | 0 | 3 | 0 | 0 | 0 |  |
| 71-♀♂ | 24 | 12 | 5 | 4 | 3 | 1 | 1 | 18 | 12 | 1 | 0 | 4 | 0 | 4 | 3 | 66.5 |
| 72-♀♂ | 0 | 1 | 0 | 0 | 0 | 0 | 0 | 0 | 0 | 0 | 0 | 8 | 0 | 0 | 0 | 57.4 |
| 73-♀♂ | 0 | 0 | 0 | 0 | 0 | 0 | 0 | 3 | 0 | 0 | 0 | 5 | 0 | 0 | 0 | 57.4 |

Note:The last four rows represent the data of male displays when females are nearby.
