# Supplementary material for: Parasite defensive limb movements enhance acoustic signal attraction in male little torrent frogs
Source: eLife. 2022 May 6;11:e76083. doi: 10.7554/eLife.76083 (PMC9122496; doi:10.7554/eLife.76083)
Supplement: Supplementary file 2. — Dyadic transition matrix of three behavioral units. [file elife-76083-supp2.docx]

**Table S2.** Dyadic transition matrix of three behavioral units.

|  | Successive behavioral unit | | | |
| --- | --- | --- | --- | --- |
|  | Call | Arm waving | Hind foot lifting | Total |
| Call | 90 | 79 | 68 | 237 |
| Arm waving | 81 | 107 | 23 | 211 |
| Hind foot lifting | 57 | 50 | 39 | 146 |
| Total | 228 | 236 | 130 | 594 |
